# Glycerol Production by Fermenting Yeast Cells Is Essential for Optimal Bread Dough Fermentation

**DOI:** 10.1371/journal.pone.0119364

**Published:** 2015-03-12

**Authors:** Elham Aslankoohi, Mohammad Naser Rezaei, Yannick Vervoort, Christophe M. Courtin, Kevin J. Verstrepen

**Affiliations:** 1 Laboratory of Systems Biology, VIB, Leuven, Belgium; 2 CMPG Laboratory of Genetics and Genomics, KU Leuven, Leuven, Belgium; 3 Laboratory of Food Chemistry and Biochemistry & Leuven Food Science and Nutrition Research Centre (LFoRCe), KU Leuven, Leuven, Belgium; University of Strasbourg, FRANCE

## Abstract

Glycerol is the main compatible solute in yeast *Saccharomyces cerevisiae*. When faced with osmotic stress, for example during semi-solid state bread dough fermentation, yeast cells produce and accumulate glycerol in order to prevent dehydration by balancing the intracellular osmolarity with that of the environment. However, increased glycerol production also results in decreased CO_2_ production, which may reduce dough leavening. We investigated the effect of yeast glycerol production level on bread dough fermentation capacity of a commercial bakery strain and a laboratory strain. We find that Δ*gpd1* mutants that show decreased glycerol production show impaired dough fermentation. In contrast, overexpression of *GPD1* in the laboratory strain results in increased fermentation rates in high-sugar dough and improved gas retention in the fermenting bread dough. Together, our results reveal the crucial role of glycerol production level by fermenting yeast cells in dough fermentation efficiency as well as gas retention in dough, thereby opening up new routes for the selection of improved commercial bakery yeasts.

## Introduction

The common brewer’s and baker’s yeast *Saccharomyces cerevisiae* limits dehydration by balancing the intracellular level of osmolytes with the extracellular water activity. Specifically in environments with lower water activity compared to the cytoplasm, cells divert part of the glycolytic flux towards the production of glycerol, while also limiting glycerol catabolism and efflux and increasing glycerol uptake from the environment [1–6].

**Fig 1:**
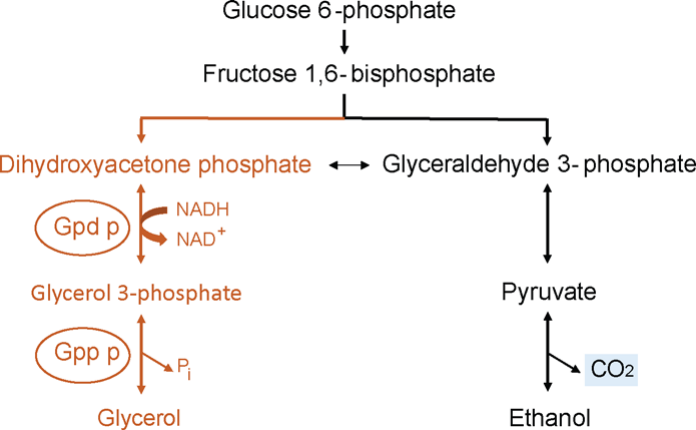
Glycerol biosynthesis and CO_2_ production. Glycerol biosynthesis from dihydroxyacetone phosphate is a two-step process. The first step which is catalyzed by the Gpd enzyme, is rate limiting for the production of glycerol. Overproduction of glycerol and carbon flux towards glycerol production is linked with decreased CO_*2*_ production from fermented sugar.

Glycerol is produced by reduction of the glycolytic intermediate dihydroxyacetone phosphate to glycerol 3-phosphate (G3P) followed by dephosphorylation of G3P to glycerol (Fig. 1). The first step of this conversion is catalyzed by NAD-dependent glycerol 3-phosphate dehydrogenase (Gpd), which is encoded as two isoforms by the genes *GPD1* and *GPD2* [7]. The expression of *GPD1*, which is required for growth at high osmolarity [8], is induced by hyperosmotic stress through the so-called HOG (High Osmolarity Glycerol) signaling pathway [9–11]. *GPD2* on the other hand, is required for glycerol production in the absence of oxygen and is believed to help maintain the cell’s intracellular redox balance [12]. Previous studies have shown that Gpd1 is the rate-limiting enzyme in glycerol metabolism [13]. Mutants with deletion or overexpression of only *GPD1* produce, respectively, less and more glycerol compared to the wild type [9,14,15]. Moreover, natural variants with different glycerol production levels often show differences in expression of *GPD1* and/or the activity of the Gpd1 enzyme [16,17].

In a previous study [18], we have shown that in the early phase of bread dough fermentation, yeast cells experience a severe osmotic shock when they are introduced in the semi-solid, high salt/sugar environment. Analysis of the transcriptome of fermenting baker’s yeast confirmed that cells activate the HOG pathway and induce glycerol production after the inoculation in bread dough [18]. However, this diversion of carbon flux towards glycerol synthesis may also limit the cell’s capacity to produce CO_2_, which is crucial for dough leavening (Fig. 1). This so-called “yeast gassing capacity” has been shown to depend on dough formulation, specific fermentation parameters, yeast physiological phase [19] and especially on intrinsic characteristics of each baker’s yeast strain. In this study we investigate the direct relation between glycerol production level and performance of yeast in bread dough and sweet dough. To specifically investigate the effect of glycerol production, we constructed deletion and overexpression mutants of the *GPD1* gene in a laboratory reference strain as well as in a commercial bakery strain. Comparison of the fermentation performance of these mutants in bread dough suggests that even a small decrease in glycerol production results in decreased bread dough fermentation capacity. In contrast, increased glycerol production leads to a swift start of fermentation and also better gas retention in sweet dough, indicating that glycerol production is a crucial property of baker’s yeast and that the positive effects of glycerol production outweigh the putative negative effects associated with decreased carbon dioxide production.

## Materials and Methods

### Strains, plasmid and microbial procedure

Two background *Saccharomyces cerevisiae* strains, a commercial bakery strain (Y243) and a laboratory strain namely FY5 (Mat α, derived from the S288C background), were used to generate mutants. In each background, we created a mutant with deletion of *GPD1* (Δ*gpd1)* and another mutant with overexpression of *GPD1* (OE-*GPD1*). *Escherichia coli* DH5α was used for cloning experiments. *E*. *coli* cultivation and media were performed as described previously [20].

The Δ*gpd1* mutants were made by first PCR-amplifying an antibiotic resistance marker surrounded by Lox sites, followed by transformation of the construct into the yeast to delete all copies of *GPD1* in the genome. Primer sequences are listed in S1 Table and we used Cre-Lox technology in order to be able to remove the selection markers. For the commercial bakery strain, four copies of *GPD1* were present in the genome and we used four different sets of primer with slightly shifted annealing target sequence to delete different copies. Deletion was confirmed by PCR (primers are listed in S1 Table).

In order to overexpress *GPD1*, the multicopy vector pVT100U-ZEO-*GPD1* carrying *GPD1* under the control of the *ADH1* promoter and terminator was introduced in yeast cells [14,21,22]. Transformants were selected on YPD medium supplemented with 50 μg/ml of phleomycin. Overexpression strains were always compared to a control strain which is background strain transformed with multicopy vector pVT100U-ZEO and was grown in the same condition as mutant.

Yeast cultures were grown under optimal conditions as described previously, and standard procedures for isolation and manipulation of DNA were used [23,24]. Expression level of *GPD1* in all mutants and control strains was analyzed using qPCR (primers are listed in S2 Table). Yeast cells were harvested in exponential growth phase for RNA extraction. For dough preparation, the yeast cells were harvested at early stationary phase and washed with 1X PBS (Phosphate Buffered Saline) before inoculation into dough.

### Intracellular glycerol extraction

To measure intracellular glycerol level of yeast cells, we first extracted all intracellular molecules containing more than one alcohol function (polyols). The cells were harvested at early stationary phase and washed twice with 1X PBS. After the washes, the cell pellet was added to 3 mL boiling 0.1 mol l^-1^ TRIS/HCL buffer (pH 7.7) containing 2 mmol l^-1^ EDTA and incubated for 5 min [25]. After centrifugation at 15000g for 10 min in order to remove the cell debris, the glycerol concentration was measured using HPLC. The extraction was performed for two biological replicates.

### Dough preparation, and fermentation

Commercial flour obtained from Ceres-Soufflet (Brussels, Belgium) was used throughout this study. Dough was prepared according to the straight-dough method using the following formula for dough with 6% sugar: 100.0 g flour (on a 14% moisture basis), 6.0% (wt wt^-1^) sucrose, 1.5% (wt wt^-1^) sodium chloride, 52.0% (vol wt^-1^) water, and 5.3% (wt wt^-1^) fresh yeast pellet (16.0±0.5% dry matter) [26]. All doughs were prepared in duplicate. In salt-free dough, salt was removed from this formula.

For sweet dough, we added 18% sugar to the dough with the following formula: 100.0 g flour (on a 14% moisture basis), 18.0% (wt wt^-1^) sucrose, 1.5% (wt wt^-1^) sodium chloride, 45.0% (vol wt^-1^) water, and 5.3% (wt wt^-1^) fresh yeast pellet (16.0±0.5% dry matter) yeast (AACC, 2000). All doughs were prepared in duplicate.

The ingredients were mixed in a 100-g pin bowl mixer (National Manufacturing, Lincoln, NE, USA) for 3 min 50 s. Next, the dough was divided into 15 ± 1 g pieces of which one piece was used for gas production measurement in a Risograph for 180 min. One pieces for immediate metabolite extraction as time 0 sample and three other pieces were fermented in a fermentation cabinet at 30°C with a relative humidity of 90% for 60, 120 and 180 min before metabolite extraction. This was done for all the mutants and wild types. The experiment was performed in two biological replicates.

### Gas production measurement

The volume of gas produced by different strains and mutants during dough fermentation was measured by using a Risograph instrument (National Manufacturing, Lincoln, NE, USA). Balls of dough were made as described above and were left to ferment for a maximum of 180 min at 30°C in the Risograph instrument. Gas production was measured continuously at 1-min intervals.

### Metabolite extraction from dough and HPLC analysis

For the measurement of glycerol concentration in fermented dough, metabolite extraction was performed by blending the dough with deionized water (with volume two times the weight of the dough at the time point we stop the fermentation) for 30 s (Waring 8011E blender, Waring Products, Torrington, CT, USA). The resulting batter was centrifuged at 11,000 g for 3 min. The supernatant was filtered using a Millex-HP 0.22 mm polyethersulfone membrane (Millipore, Carrigtwohill, Ireland) and immediately stored at −20°C until further analysis.

The concentration of glycerol in the extraction was measured with ion-exclusion high performance liquid chromatography (HPLC) using a LC-20AT modular HPLC system (Shimadzu, Kyoto, Japan) connected to a RID-10A refractive index detector (Shimadzu). Separation was carried out using an ion-exclusion ROA-organic acids guard (50 × 7.8 mm) and analytical (300 × 7.8 mm) column (Phenomenex, Torrance, CA, USA) and a mobile phase consisting of 2.50 mM H_2_SO_4_ at a flow rate of 0.60 mL/min. The guard and analytical column were kept in an oven at 60°C. Analyses were performed on two replicates.

### Rheofermentometer analysis of fermenting dough

The Rheofermentometer F3 (Chopin Technologies, Villeneuve-la-Garenne, France) was used to follow up fermentation characteristics of fermenting dough using the procedure explained by Czuchajowska and Pomeranz with slight modification [27].

Dough (100 g flour basis) was made as described above and placed in the basket of the Rheofermentometer. The Rheofermentometer measures dough height as a function of time using a displacement sensor. Total gas production and gas retention are measured with a pressure sensor. All tests were conducted as single measurements for 6 h at 30°C.

### Kieffer rig uniaxial dough extensibility test

The impact of glycerol on uniaxial dough extensibility was studied using Kieffer rig dough extensibility test [28,29]. For measurements, dough was prepared in a 10.0 g scale pin bowl mixer (National Manufacturing). Flour was mixed with the optimum amount of water for for 3 min 50 s. To study the effect of glycerol on dough properties, different amounts of glycerol was added to the dough to adjust the molarity of glycerol present in dough to the levels of 1.6, 3.2, 15.7 and 31.2 mmol/100 g flour. The dough was then placed in a paraffin oil coated Teflon mold and compressed with a lubricated top plate to form uniform dough strands of approximately 1 g. The strands were left to rest at 30°C for 30 min before they were used for measurements. Extension tests were performed using the Kieffer rig dough extensibility fixture (Stable Micro Systems Ltd., Surrey, UK) mounted on an Instron 3342 Single Column Testing System (Norwood, MA, USA) with a 50 N load cell. Three different doughs were prepared and measured for each concentration of glycerol. From each dough, 6 to 8 dough strands were used for analysis. The parameters derived from the resulting load—displacement curve are maximum extensibility (mm), maximum force for extension (N) and the total force needed for breakage (mJ), i.e. the area under the load—displacement curve.

### Statistical analysis

Statistical evaluation of the results was performed with SAS software 9.2 (SAS Institute, Cary, NC). One-way analysis of variation (ANOVA) was conducted to compare the differences in maximum dough extensibility, maximum force for extension and the total force required for dough breakage. Verification of significant differences was done by a Tukey test at a 5% significance level.

## Results

### Deletion and overexpression of *GPD1* changes cellular glycerol production

**Fig 2:**
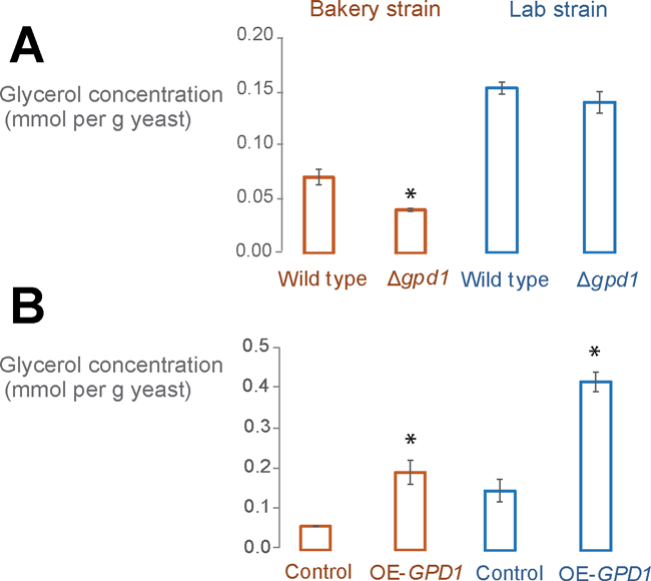
*GPD1* tunes intracellular glycerol concentration. We measured the intracellular glycerol level before exposure to the high osmolarity condition (before inoculation into dough). Deletion (A) and overexpression (B) of GPD1 in both yeast backgrounds results in respectively decreased (p < 0.05 for bakery strain) and increased (p< 0.05 for both strains) intracellular glycerol level. The control strain used in panel B is a wild-type (WT) strain transformed with the same overexpression plasmid that did not contain the *GPD* gene (“empty vector control”). Bar graphs show the average of the repeats and error bars represent the standard deviation from the mean. Bar graphs with asterisk are significantly different from their corresponding control.

To study the effect of glycerol production in fermenting yeast cells on bread dough fermentation, we generated mutant yeast strains that show either reduced or elevated glycerol production. Specifically, to reduce glycerol production, we deleted all copies of the glycerol synthesis *GPD1* gene, whereas glycerol production was elevated by introducing a multicopy *GPD1*-overexpression plasmid. We worked with two different strains, a commonly used laboratory strain (FY5, a haploid S288c descendant) and a tetraploid commercial bakery strain. In the bakery strain, deletion of all four genomic copies of *GPD1* led to a 40% reduction in intracellular glycerol levels. By contrast, deletion of *GPD1* in the haploid laboratory strain led to only a 20% decrease in intracellular glycerol levels (Fig. 2A). Overexpression of *GPD1* resulted in a 300% increase in intracellular glycerol level in both yeast strains (Fig. 2B). The expression data of *GPD1* gene for deletion and overexpression mutants in both background strains are shown in S1 Fig.

### Glycerol production is crucial for efficient dough fermentation

**Fig 3:**
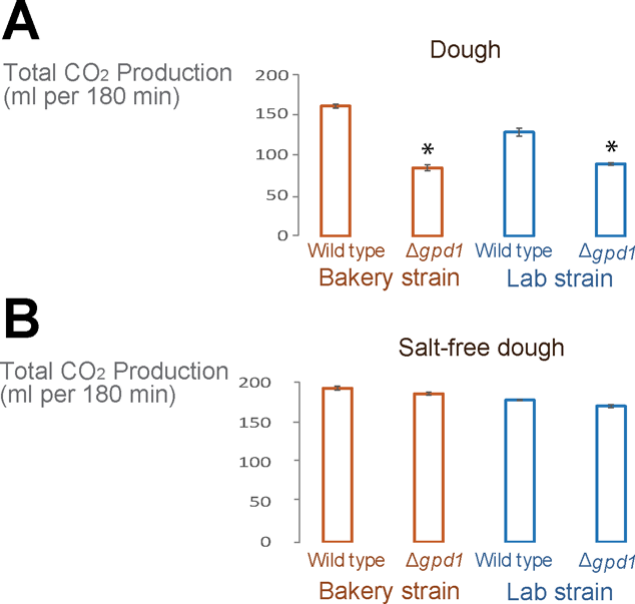
Glycerol production is crucial for efficient dough fermentation. Δ*gpd1* mutants show impaired dough fermentation (p < 0.05) (A) even though these mutants show a similar fermentation profile as the respective wild-type strains when salt-free dough is used (B). Bar graphs show the average of the repeats and error bars represent the standard deviation from the mean. Bar graphs with asterisk are significantly different from their corresponding control.

To investigate the effect of decreased glycerol production and lower initial glycerol cell content on the performance of yeast during bread dough fermentation, we used the Δ*gpd1* mutants for dough fermentation and measured CO_2_ production as proxy for fermentation capacity. Our data indicates that mutants in both backgrounds show a 30 to 50% reduction in fermentation capacity compared to the respective wild type cells (Fig. 3A). Interestingly, these mutants do not show an aberrant fermentation profiles when salt-free dough is used, suggesting that the decreased fermentation efficiency observed for normal bread dough is linked to the high osmolarity and low water activity in regular dough (water activity of 0.97, compared to 0.99 for salt-free dough) rather than being a consequence of maintaining a proper redox balance (Fig. 3B).

### Overexpression of *GPD1* improves fermentation of high-sugar dough in a laboratory strain

**Fig 4:**
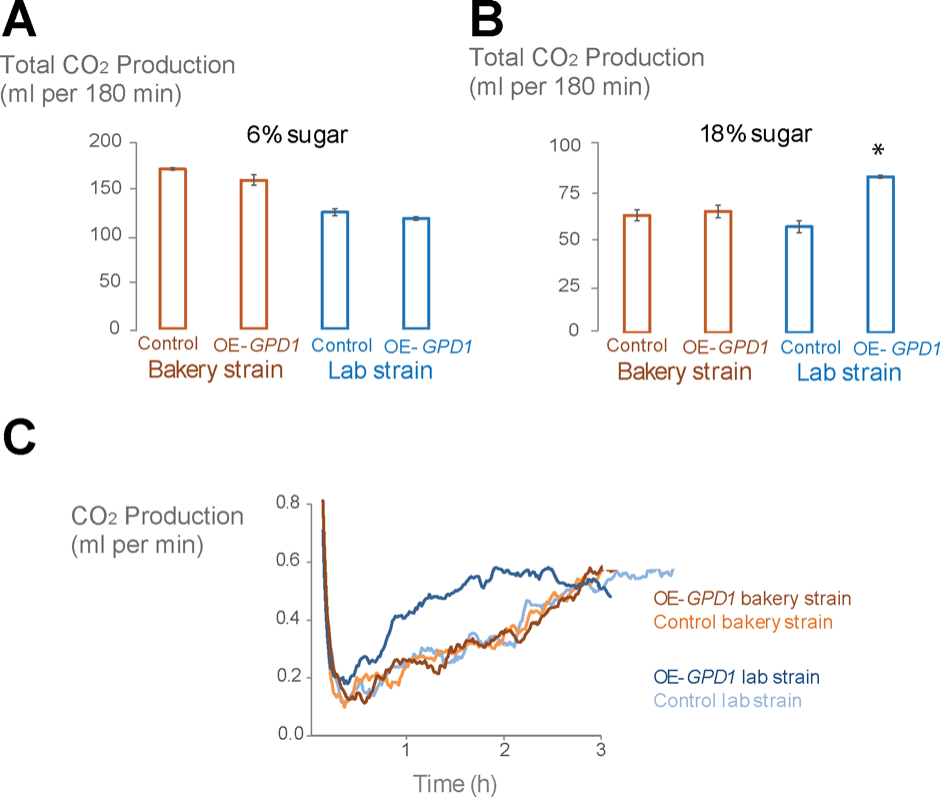
*GPD1* overexpression increases the fermentation capacity of the laboratory strain in high-sugar dough. (A) In low-sugar dough, increased glycerol production does not influence the fermentation capacity, (B) but in higher osmotic pressure, 50% improvement in one of the backgrounds was observed (p < 0.05). Bar graphs show the average of the repeats and error bars represent the standard deviation from the mean. The bar graph with asterisk is significantly different from its corresponding control. (C) CO_2_ measurement profile during fermentation of high-sugar dough (18%) recorded by Risograph shows that this improvement in the fermentation capacity of laboratory strain is due to the shorter lag of the mutant compared to the control and the swift start of fermentation.

Next, we tested the effect of higher level of glycerol production and higher initial glycerol cell content on CO_2_ production levels. To this end, we performed the experiment with two different sugar concentrations, namely in regular dough with 6% sugar (water activity: 0.97) and in high-sugar dough with 18% sugar (water activity: 0.95). In the low-sugar dough, overexpression and control strains showed comparable fermentation capacities (Fig. 4A). In the high-sugar dough, however, overexpression of *GPD1* resulted in a 50% increase in fermentation capacity compared to the control laboratory strain. In contrast, *GPD1* overexpression did not cause any significant changes in fermentation performance of the commercial bakery strain (Fig. 4B). The increased fermentation capacity of OE-*GPD1* in the laboratory strain background is due to faster adaptation to the high osmolarity and shorter lag in the onset of fermentation (Fig. 4C).

### Elevated glycerol correlates with better gas retention in dough

**Fig 5:**
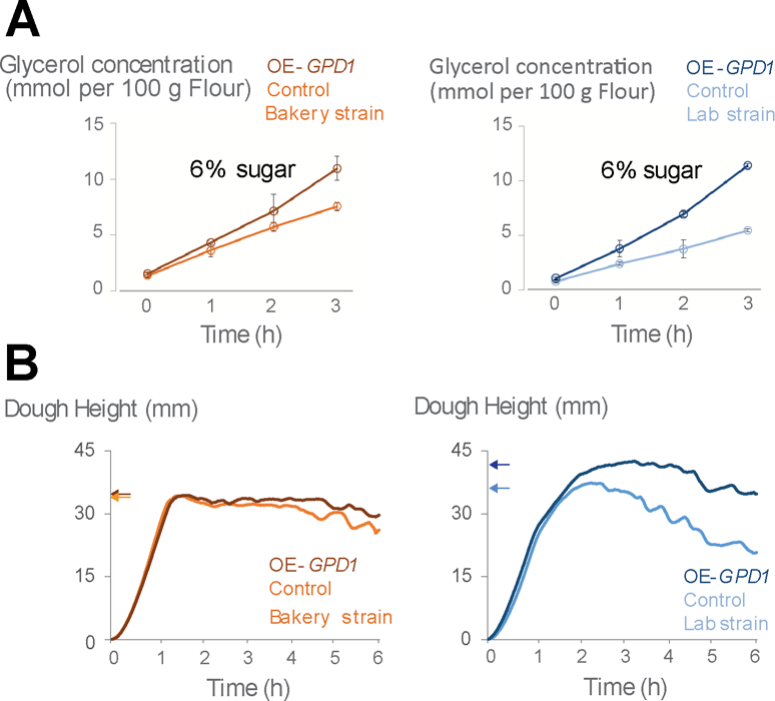
Fermenting with the strain with higher glycerol production level leads to stronger dough structure. (A) *GPD1* overexpression results in a 30% (bakery strain, left) or 100% (laboratory strain, right) increase in glycerol levels in dough. Error bars represent the standard deviation from the mean (average of the repeats). (B) Rheofermentometer analysis indicates that the dough shows better gas retention once fermented with the *GPD1*-overexpressing mutant. The arrows represent the maximum height of dough fermented with different strain or mutants. The bigger maximum height and slower decrease of the height is associated with better gas retention in dough. The difference is more pronounced in the laboratory strain (right).

In *Saccharomycess cerevisiae*, when not under osmotic stress, a big fraction of glycerol leaks to the environment [30]. Measuring glycerol levels in dough fermented with wild type or *GPD1*-overexpressing mutants confirms that some of the produced glycerol leaks into the medium, with *GPD1*-overexpressing mutants leaking 30% (for the bakery strain) to 100% (for the laboratory strain) more glycerol into the dough compared to their respective control (Fig. 5A). To investigate whether the changes in glycerol production level and the glycerol concentrations in dough might impact its development, we compared the development of the dough fermented with control strain and the dough fermented with *GPD1*-overexpressing mutant. To exclude the effect of differences in fermentation rate, and also due to the fact that in high osmolarity, cells tend to retain the glycerol, we performed this experiment only in 6% sugar where in control and overexpression mutant show similar fermentation capacities (see Fig. 4). The results show that fermentation with *GPD1*-overexpressing mutants results in improved gas retention in dough as measured by Rheofermentometer (Fig. 5B). This effect is much more pronounced in the laboratory strain where we see the dough fermented with overexpression mutant reaches higher maximum height compared to the control (Fig. 5B). Moreover, after reaching the maximum rate, the dough is more stable and loses its height (the gas) more slowly. This criteria is linked with bigger final volume of the dough. In the bakery strain, the effect of *GPD1* overexpression on the dough characteristics is much less pronounced, possibly because in the commercial strain, *GPD1* overexpression leads to only 30% increase of the already high glycerol production of the wild type strain. This is in keeping with the observation that the wild type bakery strain already produces a very stable dough that retains the carbon dioxide gas very well compared to the laboratory strain (Fig. 5B).

To confirm the positive effect of glycerol on dough rheology, different levels of exogenous glycerol was added to yeast-free dough and the Kieffer rig dough extensibility test was performed to compare rheological properties of dough with added glycerol to that of control dough. S3 Fig. shows the maximum extensibility (mm) and maximum force for extension (N). The increase in maximum dough extensibility and the decrease in maximum force for extension are both indications of dough softening. These results are in keeping with the results obtained in Rheofermentometer analysis that the increase in glycerol production leads to increased gas holding capacity of the dough. Increased glycerol content of dough leads to the dough softening and consequently, less force is required for the increase in dough height.

## Discussion

Our study reveals the effect of changes in glycerol production on bread dough fermentation. Specifically, our results demonstrate that glycerol production level in yeast cells is important for dough fermentation efficiency and level of gas retention in dough, and that the glycerol-3-phosphate dehydrogenase Gpd1 plays a central role in glycerol synthesis during bread dough fermentation. Since the effect of glycerol production disappears in salt-free dough that has a much higher water activity compared to normal dough, it seems likely that, rather than an effect of problems with the cellular redox balance, the effect of glycerol production is linked to the efficiency with which yeast cells can adapt to the osmotic stress in dough.

Interestingly, *GPD1*-overexpression leads to a much larger increase in high-sugar dough fermentation efficiency in the laboratory strain compared to the commercial bakery yeast strain. This result is consistent with the finding of a previous study that reports that in some strains there is no correlation between osmotolerance and high glycerol levels or another study that explains that the domestication of bakery yeasts has enhanced the maltose fermentation without a similar effect on the ability to ferment under high osmotic stress [31,32]. Our findings also corroborate and help to explain previous studies that have reported increased fermentation performance of yeast strains that were pre-exposed to osmotic stress or to exogenous glycerol before inoculation into bread dough [33–35].

In some industrial processes such as wine fermentations, increased glycerol production is linked to a pronounced decrease in carbon dioxide and ethanol production [14,21,36,37]. However, in the case of bread dough fermentation, the diversion of part of the carbon flux to glycerol does not seem to tip the overall balance and results in an increased fermentation rate, presumably because the cells are coping better with the high osmolarity of the medium.

Perhaps most importantly, glycerol production level does not only seem to be vital for efficient dough fermentation, but also for optimal dough development and carbon dioxide retention. Again, this effect was less pronounced in the commercial bakery strain compared to the laboratory strain, presumably because the gas retention in the dough fermented by the former is already much better than the dough fermented by the laboratory strain. This might be due to the fact that the commercial bakery strain produces higher level of glycerol compared to the laboratory strain (See Fig. 5A) or due to many other differences between the two strains. Although it’s been reported by several authors that glycerol can impact the sensory profile and ultimate firmness of bread after storage [38,39], no study has explained the impact of glycerol production level on rheological properties of dough to the best of our knowledge. It is important to note, however, that increased glycerol production leads to changes in the metabolic fluxes resulting in changes in production level of different organic acids [40] (Changes in production of acetic acid and succinic acid are shown in S2 Fig.) which can impact the rheological properties of dough. For example, addition of succinic acid in the levels produced by the yeast during dough fermentation to the dough formulation showed decrease in dough extensibility and increase in the maximum force required for dough breakage, which is the opposite of the observation with addition of glycerol [41]. The rheological changes observed in the dough fermented by mutants are most probably result of the accumulative effect of changes in the levels of various metabolites produced by the yeast.

Together, our data suggest that a high glycerol production level contributes to optimal dough fermentation and dough development. This implies that it may be interesting to explore the potential of selection of natural yeast species or variants that show high glycerol production, such as *S*. *kudriavzevii*, for dough fermentation. Another possible route that could be explored is the generation of interspecific hybrids between strains or species with elevated glycerol production and existing commercial baking yeasts. This could also yield strains that may be interesting for use in frozen dough, since previous studies show that cryophilic or cryotolerant strains produce more glycerol than non-cryotolerant yeasts [42–45]

## Supporting Information

S1 Fig Changes in gene expression as a result of deletion or overexpression of *GPD1* gene (compared to the respective control). (TIF)

S2 Fig Elevated glycerol production in mutants, results in increased acetic acid production and decreased succinic acid production. (A) *GPD1* overexpression results in a 13% (bakery strain, left) or 12% (laboratory strain, right) increase in acetic acid levels in dough. Error bars represent the standard deviation from the mean (average of the repeats). (B) Succinic acid levels decreased in both cases (22% decrease in bakery strain and 43% in laboratory strain).
(TIF)

S3 Fig Addition of exogenous glycerol leads to changes in the rheology of dough. Different levels of exogenous glycerol was added to yeast-free dough and the Kieffer rig dough extensibility test was performed to compare rheological properties of dough with added glycerol to that of control dough. The increase in maximum dough extensibility and the decrease in maximum force for extension are both indications of dough softening which results in improved gas retention. Asterisk indicates significant difference between sample and the control (Tukey HSD test, p < 0.05).
(TIF)

S1 Table List and sequence of primers that were used for deletion of GPD1 (long sequences) or deletion confirmation (short sequences). (DOCX)

S2 Table List and sequence of qPCR primers. (DOCX)
