# Supplementary figures and images for: Glycerol Production by Fermenting Yeast Cells Is Essential for Optimal Bread Dough Fermentation
Source: PLoS One. 2015 Mar 12;10(3):e0119364. doi: 10.1371/journal.pone.0119364 (PMC4357469; doi:10.1371/journal.pone.0119364)

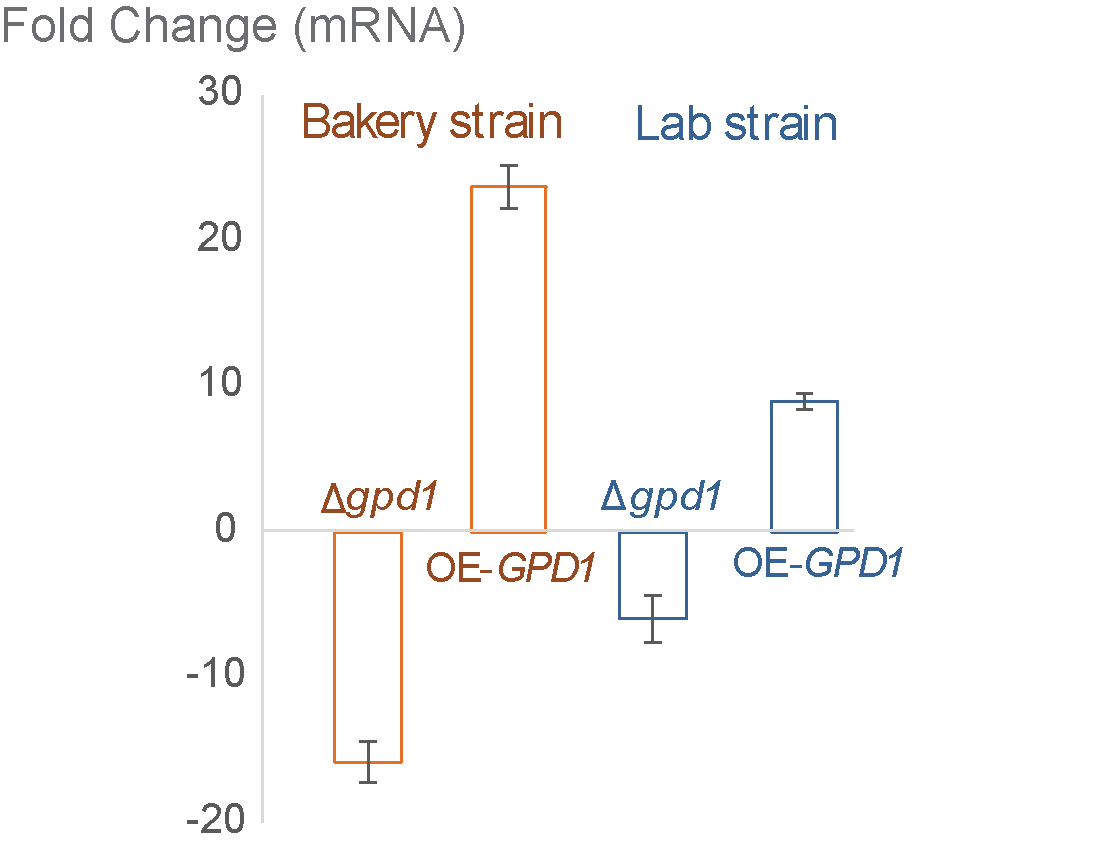

Supplement: S1 Fig — (TIF) [file pone.0119364.s001.tif]

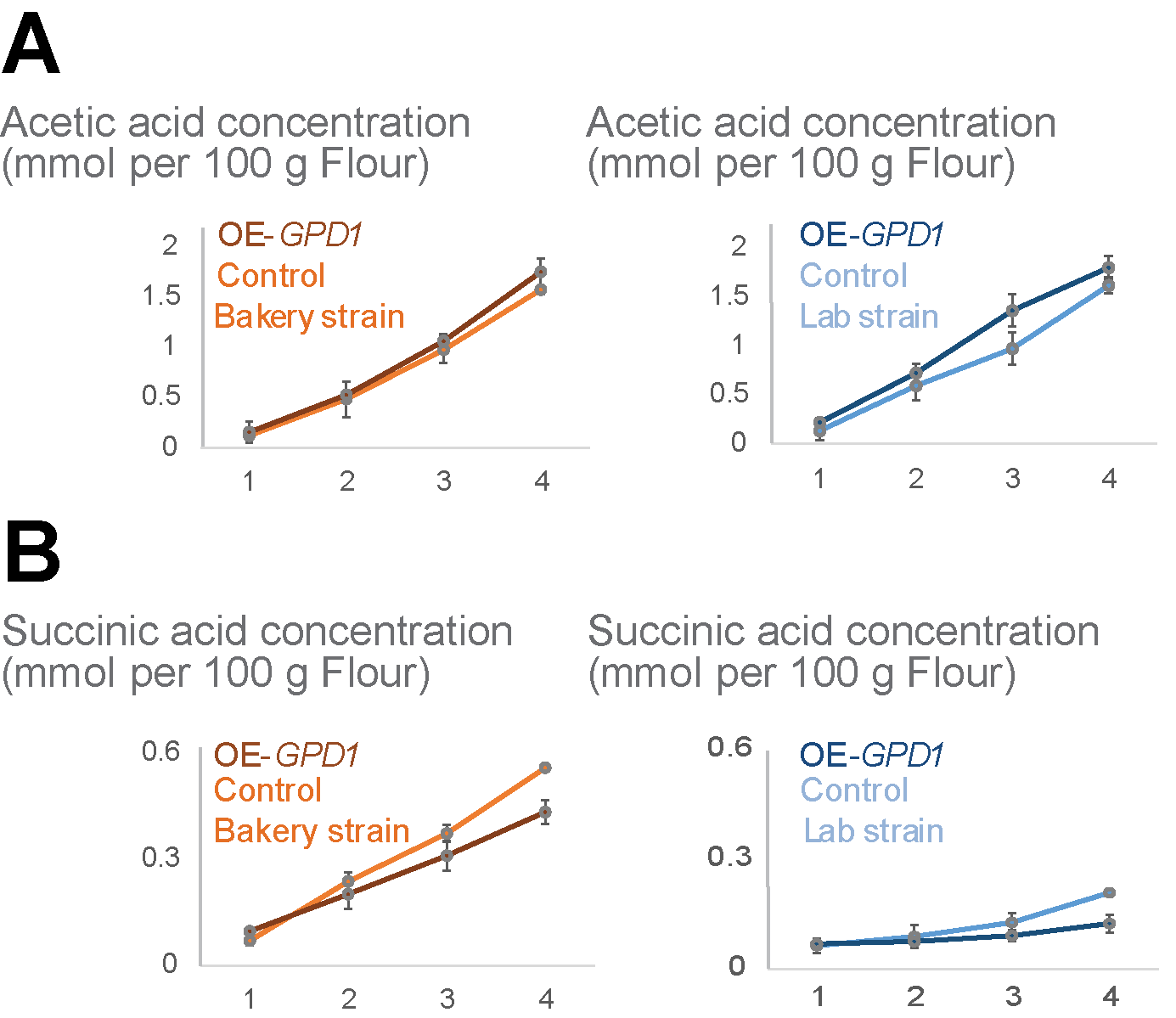

Supplement: S2 Fig — (A) GPD1 overexpression results in a 13% (bakery strain, left) or 12% (laboratory strain, right) increase in acetic acid levels in dough. Error bars represent the standard deviation from the mean (average of the repeats). (B) Succinic acid levels decreased in both cases (22% decrease in bakery strain and 43% in laboratory strain). (TIF) [file pone.0119364.s002.tif]

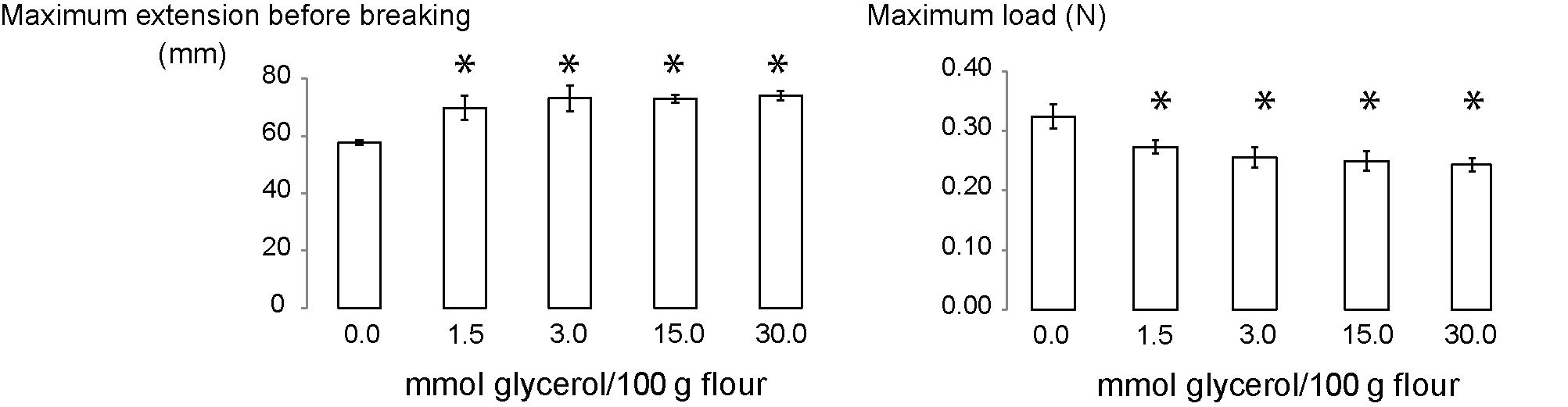

Supplement: S3 Fig — Different levels of exogenous glycerol was added to yeast-free dough and the Kieffer rig dough extensibility test was performed to compare rheological properties of dough with added glycerol to that of control dough. The increase in maximum dough extensibility and the decrease in maximum force for extension are both indications of dough softening which results in improved gas retention. Asterisk indicates significant difference between sample and the control (Tukey HSD test, p < 0.05). (TIF) [file pone.0119364.s003.tif]
