# Supplementary material for: Glycerol Production by Fermenting Yeast Cells Is Essential for Optimal Bread Dough Fermentation
Source: PLoS One. 2015 Mar 12;10(3):e0119364. doi: 10.1371/journal.pone.0119364 (PMC4357469; doi:10.1371/journal.pone.0119364)
Supplement: S1 Table — (DOCX) [file pone.0119364.s004.docx]

Table S1: List of primers that was used for deletion of *GPD1* (long sequences) or deletion confirmation (short sequences).

| **Primer** | **Sequence** |
| --- | --- |
| Forward primer to delete the first copy of *GPD1* | GTAGATCAGGTCAGTACAAACGCAACACGAAAGAACAAAACAGCTGAAGCTTCGTACGC |
| Reverse primer to delete the first copy of *GPD1* | AAGGTGTGGCACTTTGTTTATTATTTAAAATACACCCATAGCATAGGCCACTAGTGGATCTG |
| Forward primer to delete the second copy of *GPD1* | GAAAACAGAAGGCCAAGACAGGGTCAATGAGACTGTTGTCCAGCTGAAGCTTCGTACGC |
| Reverse primer to delete the second copy of *GPD1* | ACGGACGCCAGATGCTGGAAGCAACTGTGCCGACAGCCTCGCATAGGCCACTAGTGGATCTG |
| Forward primer to delete the third copy of *GPD1* | TGTCCCTATGTCTCTGGCCGATCACGCGCCATTGTCCCTCCAGCTGAAGCTTCGTACGC |
| Reverse primer to delete the third copy of *GPD1* | AGTGGTGTTGTAACCACCCTCAAAAAATGCGGAAGAGGTGCATAGGCCACTAGTGGATCTG |
| Forward primer to delete the forth copy of *GPD1* | AACAAATCAAACACCCACACCCCGGGCACCCAAAGTCCCCCAGCTGAAGCTTCGTACGC |
| Reverse primer to delete the forth copy of *GPD1* | ACAGCTAATATTATAATGGAAAATCCTGTTGCTCTATAGGGCATAGGCCACTAGTGGATCTG |
| Forward primer to check the deletion (Upstream the ORF) | TCCTCCACAAAGGCCTCTCC |
| Reverse primer to check the deletion (downstream the ORF) | GTGCACACTCAAACATATGCGC |
| Forward primer to check the deletion (in the ORF) | GGCTACTTCTGGTAAGGACG |
| Reverse primer to check the deletion (in the ORF) | ATGCGGAAGAGGTGTACAGC |
| Forward primer to check the deletion (in the ORF) | TCAATTTTTGCCCCGTATCTG |
| Forward primer to check the deletion (in the Hygromycin resistance marker) | TGGCTGTGTAGAAGTACTCG |
| Forward primer to check the deletion (in the Kanamycin resistance marker) | GTAAAAAGCCTGAACTCACCG |
