# Supplementary material for: Glycerol Production by Fermenting Yeast Cells Is Essential for Optimal Bread Dough Fermentation
Source: PLoS One. 2015 Mar 12;10(3):e0119364. doi: 10.1371/journal.pone.0119364 (PMC4357469; doi:10.1371/journal.pone.0119364)
Supplement: S2 Table — (DOCX) [file pone.0119364.s005.docx]

Table S2. List and sequence of qPCR primers.

| **Primer** | **Sequence** |
| --- | --- |
| Forward primer for *GPD1* | AAAGAAGTTCACGAATGGTTGGA |
| Reverse primer for *GPD1* | ATACGGCTTCAAATAATGGGAAGT |
| Forward primer for *ACT* | CTCCACCACTGCTGAAAGAGAA |
| Reverse primer for *ACT* | CCAAGGCGACGTAACATAGTTTT |
